# Correction

**DOI:** 10.1080/13510002.2024.2327255

**Published:** 2024-04-02

**Article title:** Sulforaphane attenuates irradiation induced testis injury in mice

**Authors:** Ran, Y., Duan, N., Gao, Z., Liu, Y., Liu, X., & Xue, B.

**Journal:** *Redox Report*

**Bibliometrics:** Volume 28, Number 01, Elocation id: 2279818

**DOI:** http://dx.doi.org/10.1080/13510002.2023.2279818

When the article was first published online, there were few errors in the Figures 2, 3, 4, and 5.

The correct figures are given below:

Also, this article had few minor changes in Figure 1 caption and Abstract. These changes do not impact the academic content of the article.

The errors have now been corrected in the online version of the article.

**Figure 2.**
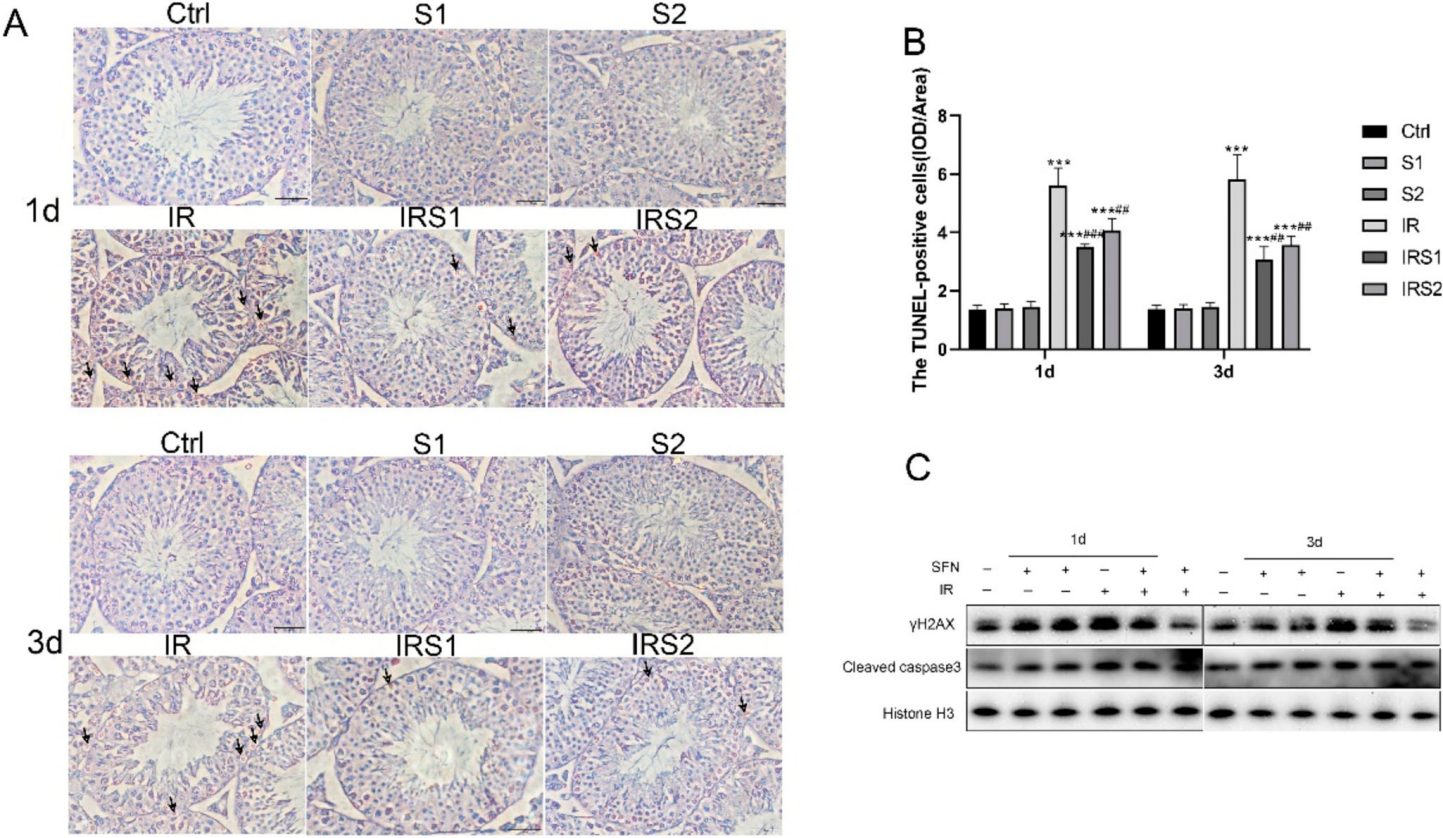
Effects of SFN on apoptosis and DNA damage of testicular germ cells after ionizing radiation. (A) The apoptosis cells were determined by TUNEL staining. Bar =50μm. →Indicates TUNEL-positive cells. (B) The TUNEL-positive cells frequency in mice testes. (C) Relative protein levels of γH2AX and Cleaved-caspase3 detected by Western blot, n = 3 for each group. * p < 0.05, ** p < 0.01, *** p < 0.001vs Ctrl; # p < 0.05, ## p < 0.01, ### p < 0.001vs IR.

**Figure 3.**
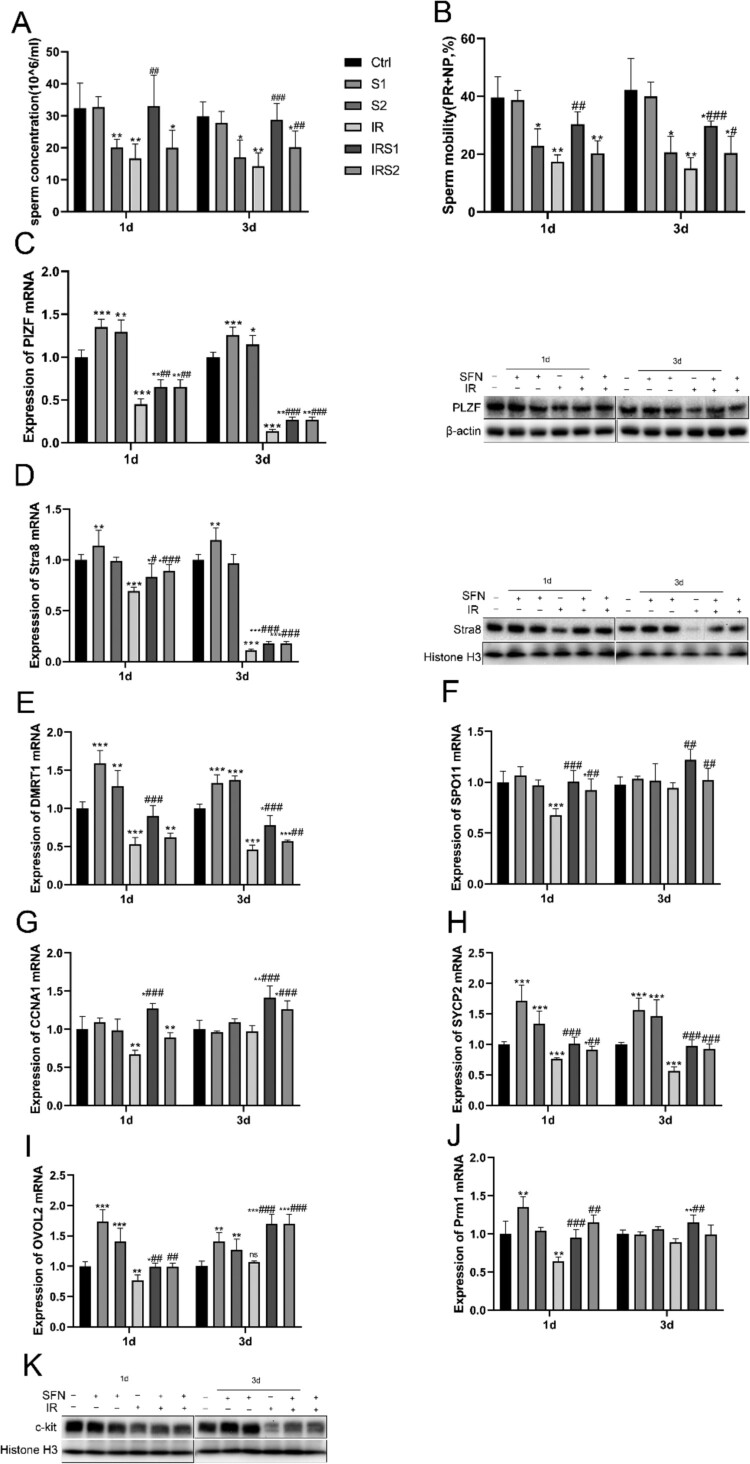
Effects of SFN on spermatogenic function after ionizing radiation. (A) Sperm concentration. (B) Total sperm motility (PR+NP). (C) Expression of PLZF mRNA and protein in testis. (D) Expression of Stra8 mRNA and protein in testis. (E) Expression of DMRT1 mRNA in testis tissue. (F) Expression of SPO11 mRNA in testis. (G) Expression of CCNA1 mRNA in testis. (H) Expression of SYCP2 mRNA in testis. (I) Expression of OVOL2 mRNA in testis. (J) Expression of Prm1 mRNA in testis. (K) Relative protein level of c-kit detected by Western blot, n = 3 in each group. * p < 0.05, ** p < 0.01, *** p < 0.001vs Ctrl; # p < 0.05, ## p < 0.01, ### p < 0.001vs IR.

**Figure 4.**
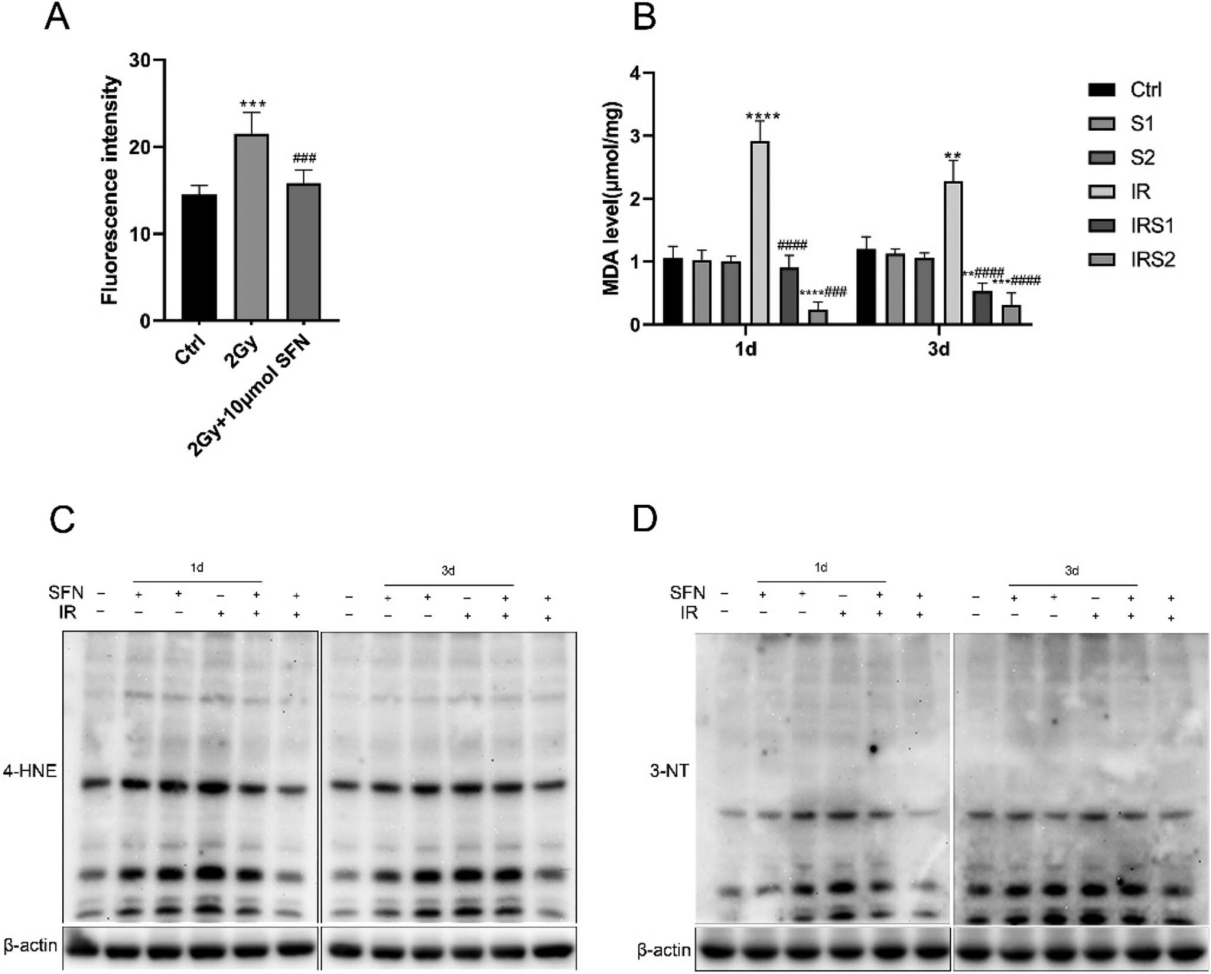
SFN inhibits ionizing radiation-induced testicular oxidative stress. (A) DCFH-DA fluorescence probe was used to detect ROS in GC-1 cells. (B) MDA level in the testis. (C), (D) Western blotting was used to detect the expression of oxidative stress indicator, 3-NT and 4-HNE. n = 3 in each group. * p < 0.05, ** p < 0.01, *** p < 0.001vs Ctrl; # p < 0.05, ## p < 0.01, ### p < 0.001vs IR

**Figure 5.**
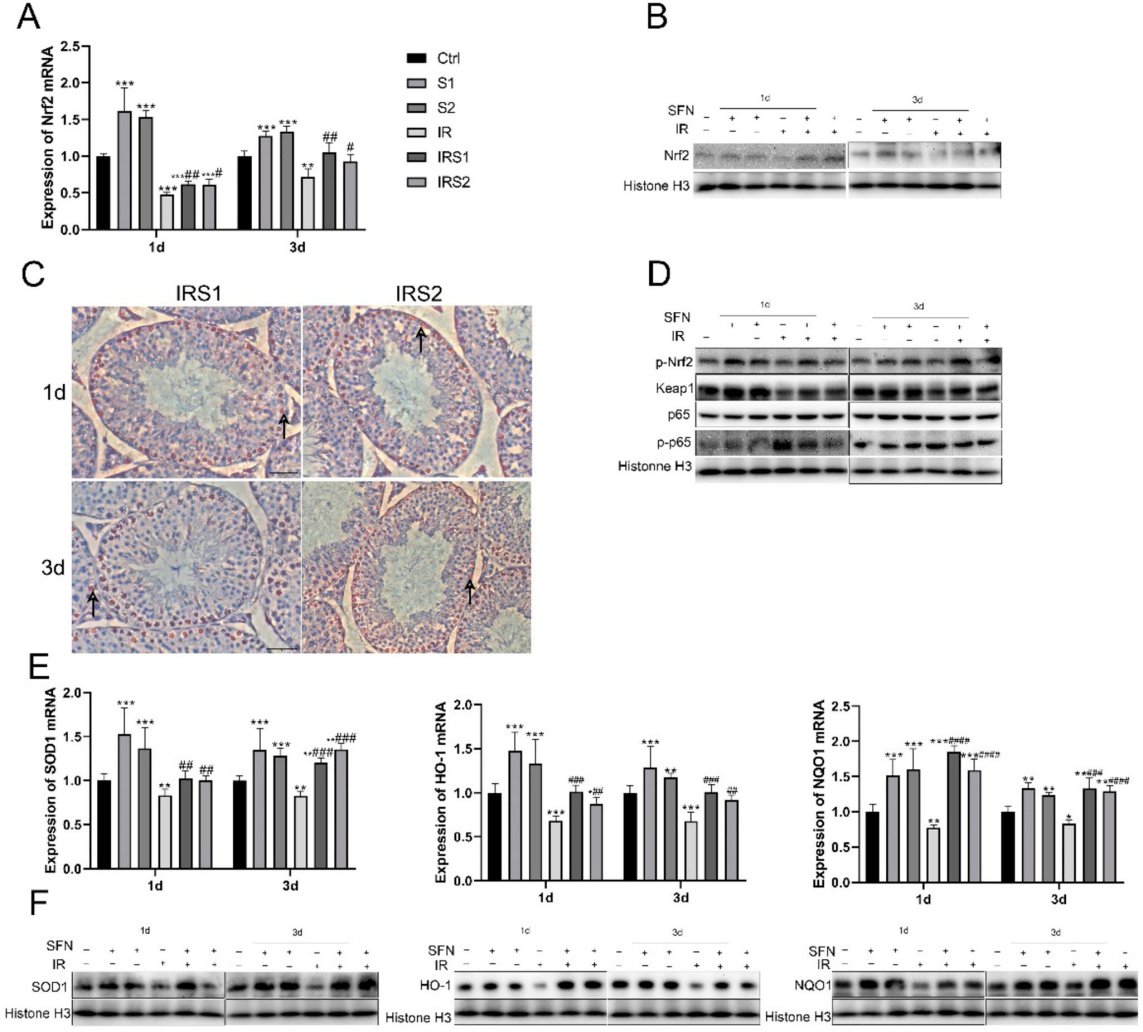
SFN upregulated Nrf2 expression and restricted NFκB activation in the testis after ionizing radiation. (A) Nrf2 expression at mRNA level was detected by real-time PCR. (B) Nrf2 expression at protein level was detected by Western blot. (C) The activation of Nrf2 was examined by immunohistochemical staining (bar =50μm). →Indicates p-Nrf2 positive cells. (D) Western blot for Nrf2 phosphorylation at Ser40 (p-Nrf2), Keap1, p65, p-p65. (E), (F) Nrf2 function was measured by determining the expression of Nrf2 downstream genes, SOD1, heme oxygenase 1 (HO-1), and NAD(P)H: quinone oxidoreductase (NQO1) at mRNA and protein levels. n = 3 in each group. * p < 0.05, ** p < 0.01, *** p < 0.001vs Ctrl; # p < 0.05, ## p < 0.01, ### p < 0.001vs IR.

